# The atherogenic index (ATH index) as a potential predictive marker of idiopathic sudden sensorineural hearing loss: a case control study

**DOI:** 10.1186/s12944-019-1016-5

**Published:** 2019-03-15

**Authors:** Anastasiya M. Kaneva, Yury K. Yanov, Svetlana G. Bojko, Olga E. Kudryavykh, Natalya N. Potolitsyna, Evgeny R. Bojko, Jon Ø. Odland

**Affiliations:** 1 Institute of Physiology of Коmi Science Centre of the Ural Branch of the Russian Academy of Sciences, FRC Komi SC UB RAS, 50 Pervomayskaya str, Syktyvkar, 167982 Russia; 2 St. Petersburg Research Institute of Ear, Throat, Nose and Speech, Ministry of Health of the Russian Federation, 9 Bronnitskaya str, St. Petersburg, 190013 Russia; 3 Medical Institute of Syktyvkar State University named after Pitirim Sorokin, Babushkina str., 11, Syktyvkar, Russia 167001; 4 Faculty of Health Sciences, NTNU, Norwegian University of Science and Technology, NTNU, NO-7491 Trondheim, Norway

**Keywords:** Sensorineural hearing loss, Lipids, apoB/apoA-I ratio, Atherogenic index of plasma (AIP), Atherogenic index (ATH index)

## Abstract

**Background:**

The importance of blood lipids in the pathogenesis of sudden sensorineural hearing loss (SSNHL) is widely discussed in the literature. However, the published results that hyperlipidaemia causes hearing problems are contradictory. The objective of this study was to establish whether increased lipid levels affect the risk of idiopathic SSNHL.

**Methods:**

A case-controlled study was conducted of 27 patients with idiopathic SSNHL and 24 healthy control subjects. All of the subjects underwent complete audiological examination. The plasma levels of total cholesterol (TC), triglycerides (TG), high-density lipoprotein cholesterol (HDL-C), apolipoprotein (apo) A-I, apoB and apoE were measured with commercially available kits (Chronolab Systems, Spain). Several clinical ratios and indices of lipid metabolism were calculated.

**Results:**

Detailed analysis of lipid metabolism in patients with idiopathic SSNHL has shown that disturbances in auditory function are associated with increased atherogenicity of the lipid profile. However, there were no significant differences in the conventional parameters of lipid metabolism (TC, TG and HDL-C) between patients with idiopathic SSNHL and subjects in the control group. Higher values of the apoB/apoA-I ratio, atherogenic index of plasma (AIP) and atherogenic index (ATH index) in patients with SSNHL indicated increased atherogenicity of the lipid profile. Binary logistic regression analysis showed that of these three indices, only higher values of the ATH index were significantly associated with an increased risk of idiopathic SSNHL.

**Conclusions:**

The ATH index can be used as a marker indicating the risk of idiopathic SSNHL when the conventional lipid indices are still normal.

## Background

Sudden sensorineural hearing loss (SSNHL) is typically defined as a rapid hearing loss of at least 30 dB in 3 contiguous audiometric frequencies within 3 days [1]. It is characterized by sudden onset, and, within a few hours, it reaches its maximum peak. SSNHL can have varying causes and aetiologies. It is estimated that a cause is identified in only 10% of patients with SSNHL [2]. The diagnosis of idiopathic SSNHL can be made definitively when no causes are found [3]. To date, there have been several proposed mechanisms for idiopathic SSNHL, which include viral infections [4]; immune-mediated mechanisms [5]; damage, including noise trauma [6]; inflammatory events [7]; otologic and metabolic diseases [8]; ototoxic chemicals and drugs [9]; head trauma [10]; neoplasms [11]; and vascular disturbances [3]. Vascular abnormalities are one of the two most common theories for the aetiology of idiopathic SSNHL; the other is inflammatory reactions (most often viral) [12]. According to vascular aetiology theories, the sudden loss of hearing could result from an acute vascular haemorrhage, occlusion by emboli, vascular diseases, vasospasms, or changes in blood viscosity [4]. Vascular damages may result in cochlear ischaemia and hypoxia [13].

The cochlea is an end organ, which is metabolically dependent on a nutrient and oxygen supply to maintain its normal physiological function. It is very sensitive to alterations in blood circulation [14]. Cochlear ischaemia is considered to be one of the most important causes of idiopathic SSNHL [15]. Hyperlipidaemia may contribute to cochlear ischaemia due to increased blood viscosity. Increased blood viscosity may decrease inner ear blood supply and cause inner ear damage [16]. Moreover, lipid metabolic disorders can lead to lipid deposits in cochlear hair cells and damage to cochlear neural cells, followed by impeded neural transduction [17].

Associations between hearing and blood lipid levels have been the focus of scientific inquiry for more than 50 years [18, 19]. In 1965, Rosen was the first to demonstrate epidemiologically that hyperlipidaemia can be a cause of auditory dysfunction. At present, there are multiple clinical and animal studies that support a relationship between hyperlipidaemia and hearing loss. Hyperlipidaemia is frequently observed in patients with idiopathic SSNHL [20, 21]. In turn, patients with hyperlipidaemia show poor hearing levels at high frequencies [16, 22]. Moreover, hearing loss associated with hyperlipidaemia tends to improve with diet control and antilipemic therapy [23, 24]. Data from animal studies have shown that diet-induced hyperlipidaemia causes structural and functional changes in the inner ear and that these changes are associated with hearing loss in a time-dependent manner [13, 14, 25]. However, the relationship between hyperlipidaemia and idiopathic SSNHL is not universally accepted. There are findings from other studies that do not support the assertion that dyslipidaemia is a risk factor for idiopathic SSNHL [26, 27].

The aim of this case-control study was to evaluate the relationship between blood lipid levels and the prevalence of idiopathic SSNHL.

## Materials and methods

### Participants

A total of 27 patients (males; mean age of 39.7 years ranging from 27 to 51 years) affected by idiopathic SSNHL were included in the study. The exclusion criteria for idiopathic SSNHL patients were as follows: acute inflammation, infection, autoimmune disorders, fluctuating cochlea-vestibular dysfunction suggestive of endolymphatic hydrops (history of vertigo with either fluctuating hearing loss, aural pressure or episodic tinnitus preceding the idiopathic SSNHL episode); a history of otologic surgery; head and/or neck trauma or barotrauma in the 10 weeks prior to idiopathic SSNHL diagnosis; otitis media; neurologic disorders that predispose a patient to deafness; recent use of ototoxic medications; neoplasm within the previous two years; or other major diseases (such as heart failure, systemic hypertension, severe lung disease, or liver or renal dysfunction). All patients had a negative history of familial deafness.

Normal subjects (24 males; mean age of 32.3 years ranging from 25 to 47 years) without a history of hearing loss or autoimmune, metabolic or circulatory diseases were included as control group. The control subjects had normal bilateral hearing (audiometric values in normal ranges according to age) and no history of cardiovascular diseases.

All participants were informed about the aim of the study and gave written informed consent. The investigation was approved by the local ethics committee and performed in accordance with the Declaration of Helsinki. This study was conducted in the Consultative and Diagnostic Center. In all subjects, systemic physical examinations, detailed otological examinations, audiological evaluations and biochemical studies were carried out.

### Hearing evaluation

All of the subjects underwent a standard evaluation that consisted of pure-tone audiometry, speech discrimination tests, impedance audiometry, tympanometry, acoustic reflex tests and magnetic resonance imaging. Pure tone and speech audiometry were carried out by an audiometer (MADSEN Itera, GN Otometrics, Denmark), and all the tests were carried out by the same speech and hearing therapist. The audiometer was installed in a sound-attenuated booth. Air and bone conduction at frequencies of 125 Hz, 250 Hz, 500 Hz, 1 kHz, 2 kHz, 4 kHz, and 8 kHz were evaluated. Audiometry was performed using a standardized protocol. Impedance audiometry was performed using an audiometer (AZ26, Interacoustics, Denmark). Tympanometry was performed to help exclude middle ear disease.

### Lipid measurements

Venous blood samples were collected from patients and control subjects after at least 12 h of fasting for the evaluation of plasma lipids. The samples were collected into vacutainer tubes containing heparin as an anticoagulant. Blood samples were centrifuged, and plasma was transferred to Eppendorf microcentrifuge tubes and stored at − 40 °C until further analysis.

Plasma total cholesterol (TC), triglycerides (TG) and high-density lipoprotein cholesterol (HDL-C) concentrations were measured using an enzymatic method with commercially available kits (Chronolab Systems, S.L., Barcelona, Spain). HDL-C concentration was determined by assaying the cholesterol in the supernatant obtained after the precipitation of apolipoprotein (apo) B-containing lipoproteins with phosphotungstate/magnesium chloride. Plasma apoA-I, apoB and apoE levels were measured using an immunoturbidimetric method with commercially available kits (Chronolab Systems, S.L., Barcelona, Spain). The samples were analysed immediately after thawing at 37 °C in a thermostatic bath. The absorbance of all samples was measured on the Powerwave 200 automated spectrophotometer (Bio-Tek Instruments, USA). The measurement of each sample was carried out in duplicate, and the mean was calculated.

Low density lipoprotein cholesterol (LDL-C) concentration was calculated according to Friedewald’s formula [28]. Several clinical indicators of lipid metabolism were computed, including the TC/HDL-C and apoB/apoA-I ratios, atherogenic index of plasma (AIP), and atherogenic index (ATH index). AIP was calculated as a logarithm of the ratio of the molar concentration (mmol/l) of TG to HDL-C (i.e., log [TG/HDL-C]) [29]. The ATH index was calculated by the following equation: ATH index = ((TC – HDL-C)*apoB)/(HDL-C*apoA-I) [30].

### Statistical analysis

All continuous variables are presented as the median and interquartile range (25th and 75th percentiles) and compared using the Mann-Whitney test. Categorical data are shown as percentages and compared using the Chi square test. Binary logistic regression was used to assess the associations between variables and idiopathic SSNHL, and odds ratios with their 95% confidence intervals were calculated. The statistical analysis was performed using Statistica 8.0 (Statsoft, Tulsa, USA). A value of *p* < 0.05 was considered statistically significant.

## Results

**Fig. 1:**
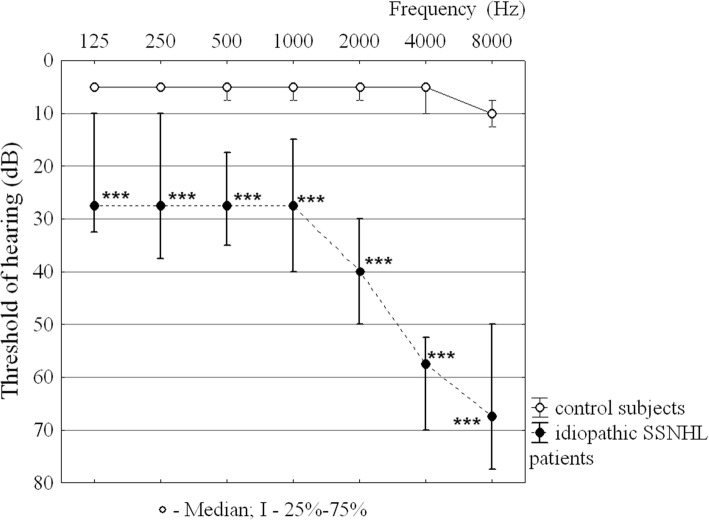
Pure-tone audiograms for idiopathic SSNHL patients and control subjects. Audiogram for control subjects denotes the average hearing thresholds of both ears. Audiogram for idiopathic SSNHL patients denotes the average hearing thresholds of the poorer hearing ear

The results of pure-tone audiometry in the study subjects are displayed in the form of an audiogram (Fig. 1). This audiogram shows that hearing in control subjects was within normal limits (5–10 dB). Patients with idiopathic SSNHL had thresholds that were 20 to 60 dB higher than the thresholds for subjects with normal hearing at all frequencies. Severe hearing loss in patients with idiopathic SSNHL was observed at high frequencies (4 kHz and 8 kHz). There is no difference between the air conduction and the bone conduction thresholds in patients with idiopathic SSNHL.

No significant differences between patients with idiopathic SSNHL and control subjects in TC, TG and HDL-C levels were observed (Table 1). The number of subjects with deviations from the reference values for TC, TG and HDL-C levels in the study groups was similar (Table 2). There were no significant differences in the calculated LDL-C concentration and TC/HDL-C ratio between patients with idiopathic SSNHL and control subjects.

**Table 1:** Plasma lipid profiles in idiopathic SSNHL patients and control subjects

Variable	Control subjects *n* = 24	Idiopathic SSNHL patients *n* = 27	*р* value ^1^
TC, mmol/L	4.09 (3.61; 4.63)	4.26 (3.84; 5.00)	0.168
TG, mmol/L	1.02 (0.79; 1.20)	1.23 (0.81; 1.76)	0.234
HDL-C, mmol/L	1.39 (1.12; 1.80)	1.36 (0.93;1.51)	0.129
LDL-C, mmol/L	1.92 (1.34; 2.91)	2.55 (1.87; 3.18)	0.199
TC/HDL-C	2.61 (2.05; 3.91)	3.59 (2.44; 4.33)	0.097
apoA-I, mg/dL	164.0 (104.0; 193.0)	120.0 (83.0; 143.0)	**0.031**
apoB, mg/dL	89.0 (70.0; 100.0)	110.0 (80.0; 162.0)	**0.048**
apoB/apoA-I	0.49 (0.42; 0.82)	1.00 (0.63; 1.57)	**0.009**
apoE, mg/dL	2.65 (1.87; 2.99)	2.43 (1.65; 3.46)	0.930
AIP	−0.17 (−0.24; − 0.06)	0.01 (− 0.12; 0.14)	**0.007**
ATH index	1.31 (0.64; 1.63)	2.63 (1.42; 3.46)	**< 0.001**

Data are presented as the median and interquartile range (25th and 75th percentile). ^1^ Mann-Whitney *(U)* test was used to estimate differences between the groups

*p*-values <0.05 are shown in boldface type

**Table 2:** Number of participants for each lipid variable defined by reference values in idiopathic SSNHL patients and control subjects

Variable	Reference range	Number of subjects		*р* value ^1^
		control subjects (*n* = 24)	idiopathic SSNHL patients (*n* = 27)	
TC, mmol/L ^a^	< 5.2	0	4 (14.8)	0.149
TG, mmol/L ^a^	< 1.7	2 (8.3)	7 (25.9)	0.202
HDL-C, mmol/L ^a^	< 1.0	1 (4.2)	7 (25.9)	0.081
LDL-C, mmol/L ^a^	< 2.6	7 (29.2)	13 (48.1)	0.272
TC/HDL-C ^b^[40]	< 4.5	3 (12.5)	6 (22.2)	0.689
apoA-I, mg/dL ^b^[41]	> 120	7 (29.2)	13 (48.1)	0.272
apoB, mg/dL ^b^[42]	< 120	2 (8.3)	11 (40.7)	**0.020**
apoB/apoA-I ^b^[43]	< 0.9	4 (16.7)	15 (55.6)	**0.010**
apoE, mg/dL ^b^[44]	> 2.7	13 (54.2)	13 (48.1)	0.882
AIP ^b^[29]	< 0.11	1 (4.2)	7 (25.9)	0.081
ATH index ^b^[30]	< 4.5	0	8 (29.6)	**< 0.033**

Data are presented as the number (percentage) of subjects. ^1^Chi-square (χ^2^) test was used to estimate differences between the groups. ^a^ Reference ranges based on the NCEP ATP III Classifications [45]. ^b^ Reference ranges taken from Refs. [29, 30, 40–44]

*p*-values <0.05 are shown in boldface type

Meanwhile, the plasma levels of apoA-I and apoB were significantly different in patients with idiopathic SSNHL compared with control subjects (Table 1). Although the apoA-I levels decreased by only 26.8% (*p* = 0.031) and the apoB concentrations increased by only 23.6% (*p* = 0.048) in patients with idiopathic SSNHL, the apoB/apoA-I ratio changed considerably. The median value of the apoB/apoA-I ratio in patients with idiopathic SSNHL exceeded the reference value and was 2 times higher than that of control subjects. The plasma levels of apoE did not differ significantly between the study groups.

Patients with idiopathic SSNHL were also characterized by higher AIP (*p* = 0.007) (Table 1), although the number of subjects with unfavourable AIP did not differ between the study groups (Table 2). The most significant difference between patients with idiopathic SSNHL and control subjects was observed for the ATH index (Table 1). The median value (25%; 75%) of ATH index in patients with idiopathic SSNHL was two times higher (2.63 (1.42, 3.46)) than that of control subjects (1.31 (0.64, 1.63)). Moreover, ATH index values higher than the reference value were observed in 29.6% of patients with idiopathic SSNHL, whereas there were no subjects with high ATH index values in control subjects (Table 2).

Binary logistic regression models were used to evaluate the associations between the risk for idiopathic SSNHL and increased values of the apoB/apoA-I ratio, AIP and ATH index (Table 3). The apoB/apoA-I ratio and AIP were not associated with idiopathic SSNHL. Meanwhile, subjects with unfavourable ATH index values had a more than 4 times higher risk of idiopathic SSNHL.

**Table 3:** Results of binary logistic regression analysis showing associations between the apoB/apoA-I ratio, AIP, ATH index and risk of idiopathic SSNHL (n = 27)

Independent variables	Odds ratio	95% Confidence interval	*p* value
apoB/apoA-I	0.91	0.17–4.91	0.908
AIP	1.53	0.02–151.8	0.851
ATH index	4.25	1.32–13.7	**0.013**

Data are presented as the odds ratio and 95% confidence interval

*p*-values <0.05 are shown in boldface type

## Discussion

The comparative analysis of plasma lipids in healthy subjects and patients with idiopathic SSNHL showed that auditory dysfunction was associated with an increase in lipid profile atherogenicity. In general, our finding is consistent with the results of other studies [16, 31]. However, these studies reported the relationship between SSNHL and the conventional lipid indices such as TC, HDL-C, LDL-C, TG and TC/HDL-C, whereas we found no significant differences between the study groups in these indices. The increase in lipid profile atherogenicity in patients with idiopathic SSNHL was indicated by a change in apoA-I and apoB levels and an increase in the apoB/apoA-I ratio, AIP and ATH index. To date, the apolipoprotein profile in patients with SSNHL has not been studied. There is only one study indicating increased apoB level in patients with SSNHL [32]. Therefore, the importance of the apoB/apoA-I ratio, AIP and ATH index as possible markers of SSNHL has not yet been established. Nevertheless, we believe that the increased values of the apoB/apoA-I ratio, AIP and ATH index in patients with idiopathic SSNHL compared with control subjects against a background of similar concentrations of traditional lipids can be considered early markers of atherogenic changes in the lipid profile. We have previously shown that the apoB/apoA-I ratio is a sensitive marker of atherogenic risk even in the subjects with normolipidaemia [33].

The effects of hyperlipidaemia on hearing function are related to the features of inner ear circulation. The cochlea is metabolically a very active organ that depends on a steady supply of nutrients and oxygen from its vasculature to maintain homeostasis [34]. At the same time, the cochlea is supplied by a terminal capillary bed and is not able to form collateral vessels, which could restore blood flow following microvascular disturbances. Therefore, the cochlea has a high sensitivity to minimal blood flow reductions, leading to tissue ischaemia and hypoxia [3, 16, 35]. In addition, cochlear blood flow depends on arterial blood pressure and cerebral circulation [36]. These features increase the risk of both functional impairments and sclerotic changes in the artery. It has been established that even a slight deterioration in the blood supply of the cochlea can lead to ischaemic damage to this organ. Histological changes in the cochlea (vascular stripe and organ of Corti) in guinea pigs were already observed after 3 months of being on a hyperlipidemic diet [37].

The negative effect of hyperlipidaemia on microcirculation lies in the fact that it affects the rheological properties of blood, the composition of membrane lipids, the protein-lipid interactions in membrane structures and the activities of membrane-bound enzymes. Moreover, with an elevation of blood lipids, lipids can attach to the surface of erythrocytes and platelets, which reduces the charge-carrying capability of erythrocytes and enhances the adhesion between cells. The increased cholesterol content in erythrocytes also affects their deformation and oxygen-carrying capacity [25, 35]. Such changes make it more difficult for the erythrocyte to travel through microcirculation. Therefore, inner ear tissues with high metabolic requirements may show altered metabolic activity in the presence of hyperlipidaemia due to reduced oxygen availability.

In our study, most patients with idiopathic SSNHL did not have hyperlipidaemia. At the same time, patients with idiopathic SSNHL exhibited higher values of the apoB/apoA-I ratio, AIP and ATH index compared with control subjects, which indicates an imbalance between atherogenic and antiatherogenic lipoproteins in the plasma and changes in lipoprotein particle size. The maintenance of the optimal balance between low density lipoproteins (LDL) and high density lipoproteins (HDL), which carry out the transport of cholesterol to peripheral tissues with its subsequent arterial internalization and reverse transport to the liver, is important in preventing atherosclerosis. With a predominance of LDL, an accumulation of LDL within the vessel wall intima begins, which leads to local oxidative modification of LDL [38]. The qualitative composition of lipoproteins also influences atherogenesis. According to the results of a prospective study, the presence of small dense LDL particles was associated with a 3.6-fold increase in the risk of ischaemic heart disease independent of LDL-C levels [39]. Thus, our results showing higher values of lipid indices in patients with idiopathic SSNHL compared with control subjects indicate the possibility of an effect of lipid profile atherogenicity on the risk of idiopathic SSNHL. Moreover, our data could explain the contradictory results regarding the association between hyperlipidaemia and hearing problems. A lack of a relationship between blood lipid levels and SSNHL was observed in studies in which the assessments of lipid profile were made by the conventional lipid indices without a detailed evaluation of atherogenicity. Whereas our results indicated a high sensitivity of the inner ear vessels to even insignificant changes in the lipid profile. This feature agrees with the view that atherosclerosis in the inner ear vessels precedes atherosclerosis in other vessels [31].

Binary logistic regression analysis showed that among the three calculated indices, only higher values of the ATH index were significantly associated with an increased risk of idiopathic SSNHL. The ATH index is a compound index of lipid metabolism and is calculated by using values of both lipids and apolipoproteins. Thus, the ATH index is an informative marker that allows an integrated evaluation of the lipid profile to be carried out. Therefore, we suggest that the ATH index can be used for the assessment of the risk of idiopathic SSNHL, since it is established that the risk of idiopathic SSNHL tends to increase as the number of cardiovascular risk factors increases [22]. Moreover, the determination of the ATH index in patients with idiopathic SSNHL will allow the selection of the adequate treatment for this pathology. Clinical studies have shown that SSNHL associated with hyperlipidaemia is treatable with timely and effective treatments [23, 24].

## Conclusion

The ATH index is a marker indicating the risk of idiopathic SSNHL. The ATH index allows us to detect abnormalities in lipid metabolism during initial stages when the conventional lipid indices are still normal. The values of the ATH index can be useful for the overall evaluation of lipid profile atherogenicity in patients with idiopathic SSNHL and choice of adequate treatments.

## Abbreviations

AIP: Atherogenic index of plasma
Apo: Apolipoprotein
ATH: Atherogenic index
HDL: High density lipoprotein
HDL-C: High density lipoprotein cholesterol
LDL: Low density lipoprotein
LDL-C: Low density lipoprotein cholesterol
SSNHL: Sudden sensorineural hearing loss
TC: Total cholesterol
TG: Triglycerides
